# Lipid peroxidation and antioxidant enzymes activity in *Plasmodium vivax* malaria patients evolving with cholestatic jaundice

**DOI:** 10.1186/1475-2875-12-315

**Published:** 2013-09-10

**Authors:** Camila Fabbri, Rita de Cássia Mascarenhas-Netto, Pritesh Lalwani, Gisely C Melo, Belisa ML Magalhães, Márcia AA Alexandre, Marcus VG Lacerda, Emerson S Lima

**Affiliations:** 1 Faculty of Pharmaceutical Sciences, Universidade Federal do Amazonas, Manaus, AM 69010-300, Brazil; 2 Institute of Biochemistry and Genetics, Universidade Federal de Uberlândia, Minas, MG 38400-902, Brazil; 3 Fundação de Medicina Tropical Dr. Heitor Vieira Dourado, Manaus, AM 69040-000, Brazil; 4 Universidade do Estado do Amazonas, Manaus, AM 69040-000, Brazil; 5 Institute of Medical Virology, Charité – Universitätsmedizin Berlin, Berlin D-10117, Germany

**Keywords:** Malaria, *Plasmodium vivax*, Antioxidant enzymes, Oxidative stress, Jaundice, Hyperbilirubinaemia

## Abstract

**Background:**

*Plasmodium vivax* infection has been considered a benign and self-limiting disease, however, recent studies highlight the association between vivax malaria and life-threatening manifestations. Increase in reactive oxygen species has already been described in vivax malaria, as a result of the increased metabolic rate triggered by the multiplying parasite, and large quantities of toxic redox-active byproducts generated. The present study aimed to study the oxidative stress responses in patients infected with *P. vivax,* who developed jaundice (hyperbilirubinaemia) in the course of the disease, a common clinical complication related to this species.

**Methods:**

An evaluation of the lipid peroxidation and antioxidant enzymes profile was performed in 28 healthy individuals and compared with *P. vivax* infected patients with jaundice, i.e., bilirubin < 51.3 μmol/L (8 patients) or without jaundice (34 patients), on day 1 (D1) and day 14 (D14) after anti-malarial therapy.

**Results:**

Hyperbilirubinaemia was more frequent among women and patients experiencing their first malarial infection, and lower haemoglobin and higher lactate dehydrogenase levels were observed in this group. Malondialdehyde levels and activity of celuroplasmin and glutathione reductase were increased in the plasma from patients with *P. vivax* with jaundice compared to the control group on D1. However, the activity of thioredoxin reductase was decreased. The enzymes glutathione reductase, thioredoxin reductase, thiols and malondialdehyde also differed between jaundiced versus non-jaundiced patients. On D14 jaundice and parasitaemia had resolved and oxidative stress biomarkers were very similar to the control group.

**Conclusion:**

Cholestatic hyperbilirubinaemia in vivax malaria cannot be totally disassociated from malaria-related haemolysis. However, significant increase of lipid peroxidation markers and changes in antioxidant enzymes in patients with *P. vivax*-related jaundice was observed. These results suggest oxidative processes contributing to malaria pathogenesis, what may be useful information for future anti-oxidant therapeutical interventions in these patients.

## Background

Malaria affects millions of people every year around the world [1]. *Plasmodium falciparum* is the most lethal species responsible for the major burden of malaria disease in Africa. However, *Plasmodium vivax* is the most abundantly distributed species worldwide. Recent reports suggest increasing clinical complications in *P. vivax* infected individuals in many endemic regions [2,3]. Brazil reports ~50% of the malarial cases in the Americas and approximately 99.5% of these cases occur in the Amazon Region [4]. Some data suggest an increased rate of hospitalization due to *P. vivax* infection in the Brazilian Amazon region over the past years [5]. Part of this increased hospitalization is related to side effects of anti-malarial drugs, such as primaquine (used as anti-hypnozoiticidal to avoid relapses), leading to haemolysis in patients with glucose-6-phosphate-dehydrogenase (G6PD) deficiency [6]. Nevertheless, a considerable proportion of patients develop clinical complications very similar to those observed in *P. falciparum* severe disease, such as jaundice, anaemia, acute renal failure, shock and coma, being the first the most common [7-10], and in most of these reports, *P. vivax* mono-infection is confirmed by PCR.

Severity criteria for *P. falciparum* are relatively well-established in the literature [11], and recently the same criteria are being used by several authors for severe *P. vivax* disease. Particularly, jaundice (impregnation of soft tissues by elevated bilirubins) in malaria may be explained by severe haemolysis (indirect bilirubin predominance) or liver cholestasis (direct bilirubin predominance). World Health Organization (WHO) most recent recommendations state that jaundice by itself (serum total bilirubins > 51.3 μmol/L) should not be used as a single marker of severity *senso strictu*, because it is not associated with higher fatality rates, unless there are other simultaneous organs dysfunctions [12,13]. Recent data from the Brazilian Amazon have also shown that hyperbilirubinaemia is not independently associated to intensive care unit hospitalization of children with vivax malaria [14]. The prognosis is favourable, and jaundice vanishes in parallel with peripheral parasitaemia clearance. However, malarial infection causing hyperbilirubinaemia with clinical jaundice leads to persistent vomiting, and is a major cause of prolonged hospitalization in many sites where *P. vivax* is endemic, contributing to increase the social and economic burden of this disease [13].

Despite the frequent occurrence of hyperbilirubinaemia, very little progress has been made in understanding the pathogenesis of cholestasis jaundice in patients with malaria, particularly in vivax disease. Increase in reactive oxygen species (ROS) has already been described in vivax malaria. As a result of the increased metabolic rate of the rapidly growing and multiplying parasite, large quantities of toxic redox-active byproducts are generated. Furthermore, a reduction in antioxidant enzymes such as glutathione peroxidase, catalase and superoxide dismutase has been observed in plasma of malaria-infected individuals [15-17]. These changes in oxidants and anti-oxidants have been associated with severe malaria in children [18]. Oxidative stress (OS) in malaria can be caused by two main mechanisms. Firstly, by the parasite, which reproduces in the erythrocytes, changing the structure and affecting parameters such as stiffness, viscosity and volume. Central to the generation of OS is the degradation of host haemoglobin by the parasite. Secondly, the OS mechanisms involve the host immune response, which initiates a cascade of defense mechanisms culminating with the release of free radicals by activated macrophages, to tackle the parasite [19,20]. Furthermore, reactive hydroxyl radicals (^•^OH) generated via mitochondrial OS, have been shown to play an important role in the liver apoptosis in a murine model of malarial infection [21,22].

Based on previous studies demonstrating the role of OS upon other clinical complications of *P. vivax* infection, it was therefore hypothesized that the transitory predominantly cholestatic jaundice seen in vivax malaria could also be associated to OS.

## Methods

### Study design

Patients with any clinical complications attributed to malaria are systematically hospitalized in the Clinical Investigation Ward of the *Fundação de Medicina Tropical Dr. Heitor Vieira Dourado* (FMT-HVD), a reference tertiary care center for infectious diseases located in Manaus (Western Brazilian Amazon). In this ward, the staff completed a standard questionnaire regarding epidemiological and clinical characteristics of the patients. Blood samples were collected before the beginning of the routine anti-malarial treatment with chloroquine (25 mg/kg over 3 days) and primaquine (0.5 mg/kg/day for 7 days), according to the National Anti-malarial Guidelines. Healthy volunteers without past history of malaria served as controls.

Patients included in this study had no diabetes or arterial hypertension history (as confirmed by fast glucose and arterial tension repeated measures throughout the hospitalization period), and were systematically phenotyped for G6PD deficiency, according to the technique described elsewhere [23]. G6PD deficient patients were not included in the analysis. In all these patients, *P. vivax* mono-infection was confirmed by PCR [24], ruling out mixed infections with *P. falciparum*. Other common infectious diseases leading to cholestasis were also ruled out through specific antibody detection (leptospirosis, hepatitis A, hepatitis B, hepatitis C and HIV), blood culture (bacterial infection), and RT-PCR (dengue virus 1,2,3 and 4). Abdominal ultrasound was also performed in all patients to exclude lithiasic cholecystitis or any other biliary tract abnormality. On day 14 (D14) after the beginning of therapy (D1), patients were informed to return to the Outpatient Clinics for clinical and laboratorial re-evaluation. Thick blood smear with parasitaemia count in 100 leukocytes, automatized full blood count and serum biochemical analysis (aspartate aminotransferase - AST, alanine aminotransferase - ALT, alkaline phosphatase - AP, gamma-glutamiltransferase – gamma-GT, bilirubins, lactic dehydrogenase - LDH) were systematically performed on D1 and D14.

### Blood samples

About 15 mL of venous blood were collected on BD Vacutainer® tubes with and without K_2_-EDTA. Aliquots of plasma were stored at −70°C before analysis.

### Oxidative stress biomarkers

Malondialdehyde (MDA) (a marker of free radical activity and lipid peroxidation) was measured using a spectrophotometer 70 UV/VIS Spectrometer PG Instruments Ltda (Beijing, China) by reaction with thiobarbituric acid (TBA) in plasma [25]. Glutathione reductase (GR; E.C. 1.6.4.2) was measured in plasma using Randox® kits on a microplate reader DTX 800 Multimode Detector, Beckman Coulter (Fullerton, CA, USA) The activity of the enzyme thioredoxin reductase (TrxR; E.C. 1.8.1.9) [26] and ceruloplasmin (CP; E.C. 1.16.3.1) [27] was performed in plasma by microplate readers. Thiol compounds were measured in plasma using the modified method [28,29] where 300 μL of 0.25 mM Tris + 20 mM EDTA pH 8.2, 3,8 μL of 5.5-ditiobis acid-2-nitrobenzoic (DTNB) 0.1 M and 7,5 μL of standard (0.5 mM glutathione) sample or water (blank) were incubated at room temperature for 15 minutes and measured in a microplate reader at a wavelength of 412 nm. All chemicals and reagents used in the study were purchased from Sigma–Aldrich® (St. Louis, MO, USA) and Randox® kits (County Antrim, UK).

### Ethical approval

The study was approved by the FMT-HVD Ethics Review Board (CAAE-0075.0.115.114-11), and all the patients signed a written consent after being informed about the objectives of the study.

### Statistical analysis

Normal distribution was assessed through Shapiro-Wilk test. Parametric data were analysed by ANOVA-one way to estimate mean differences. When significant, *post-hoc* Tukey test was performed. Kruskal-Wallis test was used for non-parametric analysis. Student and Mann–Whitney tests were used when only two groups were compared. Frequency differences were detected using chi-square. Correlations between variables were performed using the Spearman test. All tests were performed in BioStat 5.0® (Universidade Federal do Pará, Belém, Brazil) and OriginPro 8.0® (Microcal, Northampton, Massachusetts, USA), and significance was considered when p < 0.05.

## Results

During the year of 2011, 25 hospitalized patients were enrolled with confirmed microscopic diagnosis of *P. vivax* mono-infection, presenting with serum total bilirubin higher than 51.3 μmol/L (3.0 mg/dL) (direct bilirubin higher than indirect bilirubin, characterizing cholestasis). During the same period, 42 patients with non-complicated vivax malaria in the same age group, with bilirubin within the normal range, were also randomly enrolled. After ruling out other comorbidities (lithiasic cholecystitis in 4, G6PD deficiency in 2, dengue fever in 5, chronic hepatitis B in 2, chronic hepatitis C in 1, HIV in 1 and Pf/Pv mixed infection by PCR in 2), a total of eight patients with vivax-related jaundice, 34 vivax patients without jaundice and 28 healthy volunteers were included in the final analysis. No complication other than hyperbilirubinaemia was observed after detailed clinical and laboratorial screening. On D14 a clinical and laboratorial screening was performed on seven out of eight with jaundice, and 18 out of 34 patients without jaundice. None of them presented with persistent parasitaemia, clinical jaundice or laboratory hyperbilirubinaemia on D14. None of the controls on D1 referred any clinical complication in between D1 and D14. Epidemiological, haematological and biochemical data are detailed in Table 1. Jaundice was more frequent among women and those experiencing malarial infection for the first time. Haemoglobin was lower in those with jaundice, and the levels of LDH, AST and ALT were higher in this group.

**Table 1:** Epidemiological, haematological and biochemical parameters of healthy volunteers and vivax malaria patients with and without jaundice (total bilurubin > 51.3 μmol/L) on Day 1

	**Healthy volunteers (n = 28)**	***P. vivax *malaria without jaundice (n = 34)**	***P. vivax *malaria with jaundice (n = 8)**	P-value*
Age	25.4 ± 4.6	37.6 ± 15.2	33.5 ± 14.0	0.4867
Male sex (%)	32	82	38	**0.0316**
First malarial infection (%)	-	24	63	**0.0316**
Time of disease (in days)	-	6.5 ± 2.7	9.7 ± 7.9	0.3960
Peripheral parasitaemia/μL	-	2,428 ± 4,276	5,353 ± 10,598	0.3578
Leukocytes (×10^3^/μL)	6.69 ± 1.65	5.33 ± 1.59	5.41 ± 1.18	0.8903
Haemoglobin (g/dL)	13.0 ± 1.4	12.8 ± 1.8	10.8 ± 1.9	**0.0051**
Platelets (×10^3^/μL)	228.5 ± 62.0	93.7 ± 52.0	85.1 ± 54.2	0.6790
TB (μmol/L)	18.1 ± 8.5	26.3 ± 10.1	109.3 ± 81.9	**< 0.0001**
IB (μmol/L)	10.1 ± 6.2	15.7 ± 7.2	28.6 ± 15.2	0.0547
DB (μmol/L)	8.0 ± 6.7	10.6 ± 6.3	80.7 ± 79.3	**0.0003**
AST (U/L)	21.5 ± 8.9	34.5 ± 17.4	74.7 ± 67.1	**0.0488**
ALT (U/L)	16.4 ± 8.7	34.5 ± 27.0	93.0 ± 91.8	**0.0420**
AP (U/L)	57.6 ± 21.4	72.4 ± 28.0	103.9 ± 68.2	0.2408
Gamma-GT (U/L)	11.5 ± 5.5	45.7 ± 37.3	106.4 ± 92.1	0.2408
LDH (U/L)	255.6 ± 74.3	425.2 ± 188.5	609.7 ± 234.8	**0.0294**

*TB* total bilirubin, *IB* indirect bilirubin, *DB* direct bilirubin, *AST* aspartate aminotransferase, *ALT* alanine aminotransferase, *AP* alkaline phosphatase, gamma-*GT* gamma-glutamiltransferase, *LDH* lactate dehydrogenase.

*Comparison between vivax malaria patients with and without jaundice. Significant values in bold.

Continuous variables are presented as mean ± SD.

### Oxidative stress biomarkers

**Figure 1:**
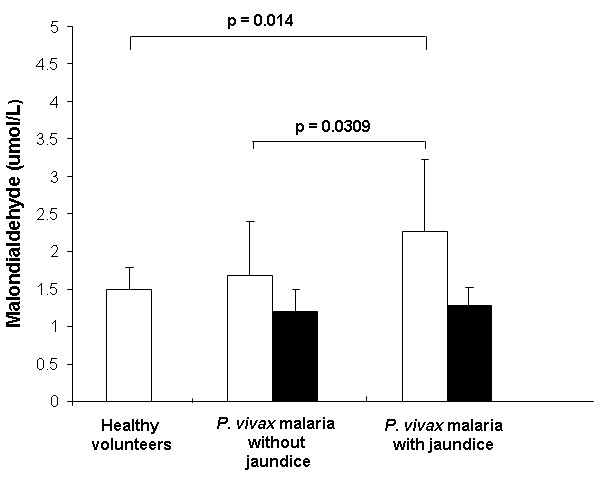
**Lipid peroxidation levels expressed as malondialdehyde (MDA) in plasma on D1 (white bars) and D14 (black bars).** Groups: Healthy volunteers (n = 28), *P. vivax* malaria without jaundice [n = 34 (D1); n = 18 (D14)] and *P. vivax* malaria with jaundice [n = 8 (D1); n = 7 (D14)]. Significant differences between the groups are indicated in each graph.

A significant increase in MDA levels on D1 in *P. vivax* malaria (with and without jaundice) group was observed compared to the control group. In addition, a significant increase of MDA was observed on D1 in the jaundiced group compared to the non-jaundiced group (Figure 1).

**Figure 2:**
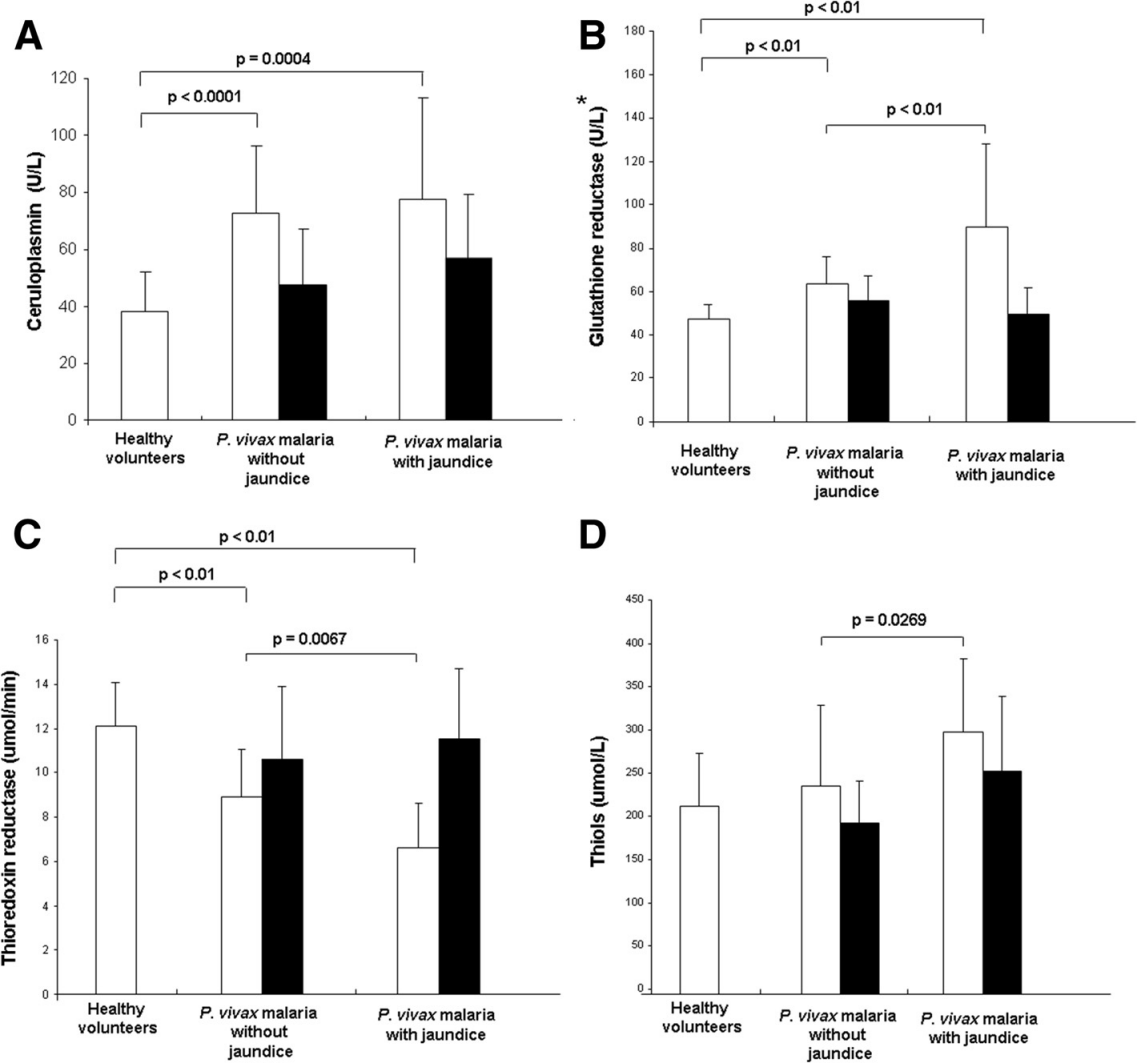
**Antioxidant enzyme activities in plasma on D1 (white bars) and D14 (black bars). (A)** Ceruloplasmin; **(C)** Thioredoxin reductase; **(D)** Thiols: healthy volunteers (n=28), *P. vivax* malaria without jaundice [n=34 (D1); n=18 (D14)] and *P. vivax* malaria with jaundice [n=8 (D1); n=7 (D14)]. Significant differences between the groups are indicated in each graph. *(B) Glutathione reductase: healthy volunteers (n=18), *P. vivax* malaria without jaundice [n=19 (D1); n=10 (D14)] and *P. vivax* malaria with jaundice [n=5 (D1); n=5 (D14)].

Figure 2 shows altered antioxidant enzyme profile in malaria patients. CP and GR are significantly increased in malaria-infected individuals (with or without jaundice) on D1 (Figures 2A and 2B) and TrxR is lower in infected patients (Figure 2C), compared to healthy volunteers. Differences in GR, TrxR and thiols between jaundiced and non-jaundiced patients are also seen (Figures 2B, 2C and 2D). On D14, markers of oxidative stress were not different from the healthy volunteers group, suggesting a convalescent state after full clinical recovery (Figure 2).

Despite of the lower level of haemoglobin in the jaundiced group, no single plasmatic oxidative stress marker was correlated with haemoglobin levels (data not shown).

## Discussion

A case series reported in 2010 showed that over 50% of patients admitted to a reference hospital in the city of Manaus had jaundice as complication for *P. vivax* malaria [8]. In the present study, evidence that *P. vivax* infection increases lipid peroxidation and alters profile of antioxidant enzymes is provided. That may eventually lead to hyperbilirubinaemia, which is not independently associated to severity [14], but was already reported in deceased patients with the clinical diagnosis of vivax malaria and other comorbidities [30]. In the eight jaundiced patients presented in this manuscript, however, further comorbidities were ruled out by detailed laboratory screening.

Despite of the very small sample size, the finding of this complication more often in females contradicts previous findings showing that male children present with more life-threatening complications [11]. No obvious explanation for this phenomenon is known, except the hypothesis that more bile duct diseases are found in women, what may contribute to the cholestatic event. Higher occurrence in patients infected for the first time suggests an adaptation mechanism that needs to be explored with bigger sample sizes and prospective study designs.

The significant increased levels of MDA in plasma of malarial patients with and without jaundice may indicate the damage triggered by free radicals produced against cell membranes, such as erythrocytes and hepatocytes [31]. Recent studies have shown increased levels of lipid peroxidation markers such as MDA, which have been implicated in a number of diseases, including malaria [16,18,31]. *Plasmodium* sp. does not have a triglyceride synthesis pathway; therefore, it obtains all the lipids from the host serum. The parasite also destabilize the cell membrane, in particular the erythrocyte membrane in order to obtain lipid [17,32].

The antioxidant enzyme TrxR activity was significantly decreased when patients with malaria were compared to the control group. TrxR catalyzes the reduction of the active site disulfide using NADPH. The high levels of ^•^NO are associated with the host-parasite interaction. TrxR was shown to be inactivated with the production of nitric oxide (^•^NO), a reactive species of oxygen found in high levels during malaria infection [33]. This may partially explain the decreased levels of this enzyme in vivax patients.

On the other hand, the antioxidant enzymes GR and CP activities were significantly increased in *P. vivax* infected patients (with or without jaundice) compared with the control group. Other studies have also demonstrated increased levels of the enzyme GR in malaria caused by *Plasmodium berghei and P. falciparum*[19]. GR is involved in maintaining an intracellular reducing environment, which is crucial to the cell in the defense against oxidative stress. Thus, increased levels of GR may be playing a role in counteracting with increased oxidant species and maintaining homeostasis [34]. Recent reports are in line with these results, confirming increased CP activity in malaria [35,36]. CP has been proposed as an important antioxidant in reducing inflammation and acute phase response by scavenging superoxide and other reactive oxygen species [37].

Thiols contain the sulfhydryl group attached to a carbon atom. They are efficient antioxidants protecting cells against consequences of damage induced by free radicals [38,39]. In this study, levels of thiol compounds were significantly increased in patients with *P. vivax* malaria with jaundice compared with *P. vivax* malaria without jaundice. Although the thiols levels in malarial patients are not significantly higher compared to the control group, results suggest that malarial patients who developed jaundice have greater oxidative stress, and thiol compounds may be trying to restore the plasmatic balance.

Several reports in the literature suggest that drugs used to treat malaria, such as chloroquine and primaquine) lead to oxidative stress, particularly in erythrocytes [40-42]. However, in this study, patients from both groups were systematically treated with these same drugs in similar dosages, as part of the national policy, allowing therefore comparability.

Bilirubin has antioxidant properties as well as pro-oxidant. At low concentrations, it acts as a scavenger of reactive oxygen species, reducing the damage caused to the cells. However, at high concentrations, as is the case of the patients with *P. vivax* malaria who developed jaundice, bilirubin has deleterious effects on tissues. It develops oxidative stress by generating intracellular ROS in hepatic cells and cause lipid peroxidation [43]. Furthermore, bilirubin can also induce apoptosis [43], complementing the information that malaria infection induces the generation of hydroxyl radical (•OH) in the liver, which may be responsible for the induction of oxidative stress and apoptosis in cells of this organ [21,22]. However, if on one side indirect bilirubin is a surrogate of haemolysis and contribute to reinforce cholestasis (jaundiced patients with lower haemoglobin levels and increase in lactate dehydrogenase support that), this compound may be faced either as a product of oxidative stress responses during malarial infection or as an inducer of oxidative stress, due to a rise in lipid and protein oxidation, ROS content, impairing glutathione metabolism (decrease of the GSH/GSSG ratio) [44]. Moreover, other studies have demonstrated that oxidative stress is increased in patients with cholecystectomy as well as in patients who developed other cholestatic diseases, and was associated with jaundice of different origin and severity [45,46].

## Conclusions

In summary, the oxidative stress in *P. vivax* patients presenting jaundice is increased. Levels of oxygen reactive species may be closely linked to the damage caused by the parasite and the subsequent release of high concentrations of bilirubin in the serum. Further studies are needed to understand the mechanisms involved in liver damage in jaundiced patients, and also to validate if similar findings are seen in other less frequent complications of *P. vivax* infection, e.g., severe anaemia, coma, acute renal failure and respiratory distress. These studies may provide further evidence for better management of *P. vivax* infections and possible future anti-oxidant supportive therapy.

## Competing interests

The authors declared that they have no competing interests.

## Authors’ contributions

CF and RCMN carried out all the biochemical analysis and drafted the manuscript, together with PL. GCM coordinated and performed all the microbiological tests. BMLM and MAAA performed the full clinical characterization of the enrolled patients. CF, MVGL and ESL participated in the design of the study. MVGL and ESL conceived of the study, and participated in its design and coordination. All authors read and approved the final manuscript.
